# Conflicting Interpretation of Genetic Variants and Cancer Risk by Commercial Laboratories as Assessed by the Prospective Registry of Multiplex Testing

**DOI:** 10.1200/JCO.2016.68.4316

**Published:** 2016-09-12

**Authors:** Judith Balmaña, Laura Digiovanni, Pragna Gaddam, Michael F. Walsh, Vijai Joseph, Zsofia K. Stadler, Katherine L. Nathanson, Judy E. Garber, Fergus J. Couch, Kenneth Offit, Mark E. Robson, Susan M. Domchek

**Affiliations:** Judith Balmaña, Hospital Vall d’Hebron and Universitat Autònoma de Barcelona, Barcelona, Spain; Laura Digiovanni, Susan M. Domchek, and Katherine L. Nathanson, University of Pennsylvania, Philadelphia, PA; Pragna Gaddam, Michael F. Walsh, Vijai Joseph, Zsofia K. Stadler, Kenneth Offit, and Mark E. Robson, Memorial Sloan Kettering Cancer Center and Weill Cornell Medical College, New York, NY; Judy E. Garber, Dana-Farber Cancer Institute, Boston, MA; and Fergus J. Couch, Mayo Clinic, Rochester, MN.

## Abstract

**Purpose:**

Massively parallel sequencing allows simultaneous testing of multiple genes associated with cancer susceptibility. Guidelines are available for variant classification; however, interpretation of these guidelines by laboratories and providers may differ and lead to conflicting reporting and, potentially, to inappropriate medical management. We describe conflicting variant interpretations between Clinical Laboratory Improvement Amendments–approved commercial clinical laboratories, as reported to the Prospective Registry of Multiplex Testing (PROMPT), an online genetic registry.

**Methods:**

Clinical data and genetic testing results were gathered from 1,191 individuals tested for inherited cancer susceptibility and self-enrolled in PROMPT between September 2014 and October 2015. Overall, 518 participants (603 genetic variants) had a result interpreted by more than one laboratory, including at least one submitted to ClinVar, and these were used as the final cohort for the current analysis.

**Results:**

Of the 603 variants, 221 (37%) were classified as a variant of uncertain significance (VUS), 191 (32%) as pathogenic, and 34 (6%) as benign. The interpretation differed among reporting laboratories for 155 (26%). Conflicting interpretations were most frequently reported for *CHEK2* and *ATM*, followed by *RAD51C, PALB2, BARD1, NBN,* and *BRIP1*. Among all participants, 56 of 518 (11%) had a variant with conflicting interpretations ranging from pathogenic/likely pathogenic to VUS, a discrepancy that may alter medical management.

**Conclusions:**

Conflicting interpretation of genetic findings from multiplex panel testing used in clinical practice is frequent and may have implications for medical management decisions.

## INTRODUCTION

Advances in next-generation sequencing technology have led to the development of multiplex panel testing for the molecular diagnosis of inherited cancer susceptibility. Commercially available panels differ in their exact composition but usually include moderate-penetrance and high-penetrance genes (with mutations reported in the literature to be associated with a relative risk [RR] of cancer between 2 and 5, or greater than 5, respectively). Genes in which mutations are associated with susceptibility to inherited cancer have been rapidly incorporated into these panels, often before robust evidence of the magnitude of the association is known. For some genes, the relatively low prevalence of mutations makes it difficult to obtain reliable estimates of the associated cancer risk.^1^

Variants of uncertain significance are frequent in panel testing and can be challenging to resolve. Despite the availability of public databases for sharing genetic variants, the development of prediction models based on protein structure and function, and the potential for laboratory-based functional analyses to determine the pathogenicity of some variants, discordant interpretation of the clinical pathogenicity of variants remains a frequent problem.^2-4^ Different standardized classification systems for interpretation of sequence-based results have been developed.^5,6^ The ClinVar database^7,8^ is a publicly available database that has allowed clinical laboratories to submit their identified variants and share their interpretation. All accessions of the same genetic variant provided by different submitters are maintained in ClinVar, which allows tracking of the changes and sharing of the evidence used for interpretation. However, submission to ClinVar is voluntary and not all laboratories choose to submit data. Moreover, resolution of the diverse submissions is a voluntary activity for experts and a Herculean task. From a clinical perspective, it is relevant to quantitate the frequencies and describe the patterns of variants with conflicting interpretations, particularly those that may impact medical management. Unrelated individuals or even members from the same family with the same genetic variant tested by different clinical laboratories may be given a different clinical assessment of variant pathogenicity. The ordering provider may not be aware that a different laboratory testing a patient’s relative has provided a different interpretation. It is crucial to identify variants with conflicting interpretations among laboratories, the frequencies and types of discrepancies, and the underlying reasons for these discrepancies to enable specific guidance for variant curation and to increase the consistency of variant interpretation among the laboratories.

Here, we describe the frequencies and types of genetic findings with conflicting interpretations in non-*BRCA* genes tested as part of panels assembled by Clinical Laboratory Improvement Amendments–approved commercial laboratories. Data were collected from individuals who underwent clinical testing and enrolled in a prospective registry. This registry includes patients with results from some commercial laboratories that currently do not deposit data in ClinVar.

## PATIENTS AND METHODS

Overall, 1,191 individuals tested for cancer susceptibility genes as part of commercial multiplex panels self-enrolled in the ongoing Prospective Registry of Multiplex Testing (PROMPT) between September 1, 2014, and September 30, 2015, the period for which data were ascertained for this analysis.

The PROMPT registry partnered with several clinical laboratories, including Ambry Genetics, Color Genomics, GeneDx, Invitae, Myriad Genetics, Pathway Genomics, and Quest Diagnostics, to recruit individuals who had undergone genetic panel testing and had at least one variant in their report. Participating laboratories advertised PROMPT within the packet of test report forms sent to individuals. Health-care providers also were educated about PROMPT at academic and industry meetings, and through e-mails. An informational Web site and video were created for participants and providers. An informational article was communicated via the Dr Susan Love Research Foundation e-newsletter to individuals enrolled in the Army of Women. Some participants found the study directly through Internet.

An enrollment site for PROMPT was built on a platform maintained by a partner organization, PatientCrossroads. To enroll, participants created an account with PatientCrossroads and consented to participate in an online genetic registry in either an identifiable (contact information available to research team) or de-identified (contact information not available to research team) manner. All participants completed the baseline questionnaire with personal and family cancer history of cancer, genetic testing, and demographics. Participants were given the opportunity to upload their genetic testing report to the PatientCrossroads portal or to send it directly to PROMPT registry staff.

**Fig 1.**
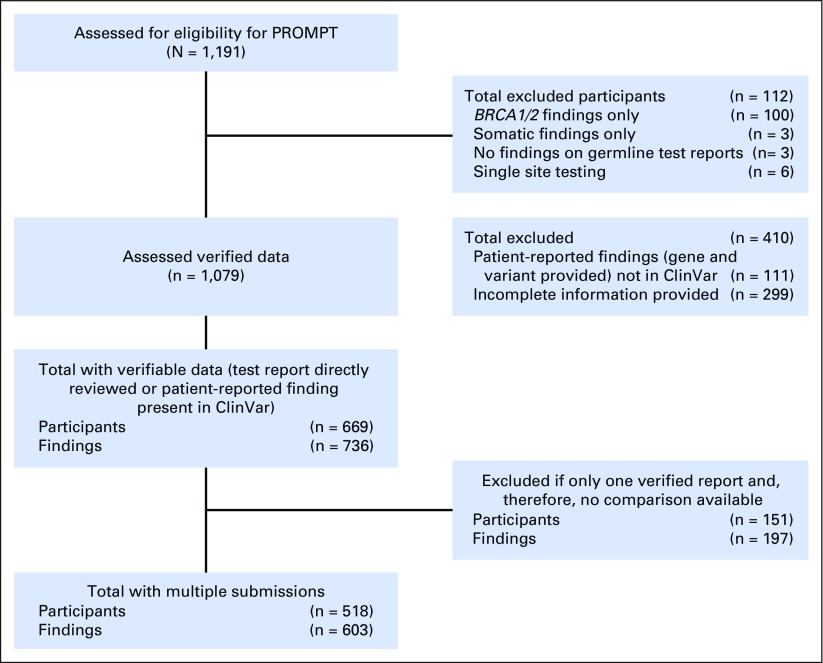
CONSORT diagram showing the flow of participants and genetic variants per participant from the PROMPT registry until inclusion for current analysis. PROMPT, Prospective Registry of Multiplex Testing.

From the initial 1,191 participants assessed for eligibility, individuals considered for this analysis were required to have verifiable genetic data by PROMPT staff (ie, a copy of a test report submitted, or a detailed self-reported test result in the registry that was found in ClinVar). Participants who had undergone tumor testing, who did not have any genetic findings, or whose unique finding was a *BRCA* result, were excluded (n = 112), as were individuals with self-reported results for which a ClinVar submission or test report was not available for confirmation (n = 410). From the 669 remaining participants, 518 (43%) had results interpreted by more than one laboratory (including at least one in ClinVar) or findings with multiple submissions reported in ClinVar, and these participants were used as the final cohort for the current analysis (Fig 1). Because commercial laboratories are not required to submit to ClinVar, if there were known differences in classification of a genetic test result among laboratories observed through reporting in the PROMPT registry, then this result was classified as a conflicting interpretation of pathogenicity. That is, a patient may have provided a report from a non-ClinVar submitting laboratory and the same finding may have been entered into ClinVar by another laboratory.

All genetic test results were checked in the ClinVar public archive (http://www.ncbi.nlm.nih.gov/clinvar/) and their clinical significance was assigned according to the submissions by different laboratories through clinical testing or submissions from research and literature curation. In ClinVar, if differences in interpretation among submitters are observed, the genetic test results are classified as conflicting interpretation of pathogenicity.^7^ A search for any update in reclassification was performed at the time of data analysis lock, with no change. Descriptive statistics were used to describe the study population.

## RESULTS

Overall, 518 participants enrolled into PROMPT were considered eligible for this analysis. Their median age was 52 years (range, 44-61 years) and 95% were female. Overall, 427 (82%) were white, and 350 (68%) had a cancer diagnosis, mostly breast cancer (n = 188; 36%). Thirty-one percent had multiple primary tumors. A total of 419 participants (81%) reported being invited into PROMPT by their health-care provider or by the laboratory where the testing had been performed (Table 1).

**Table 1.**
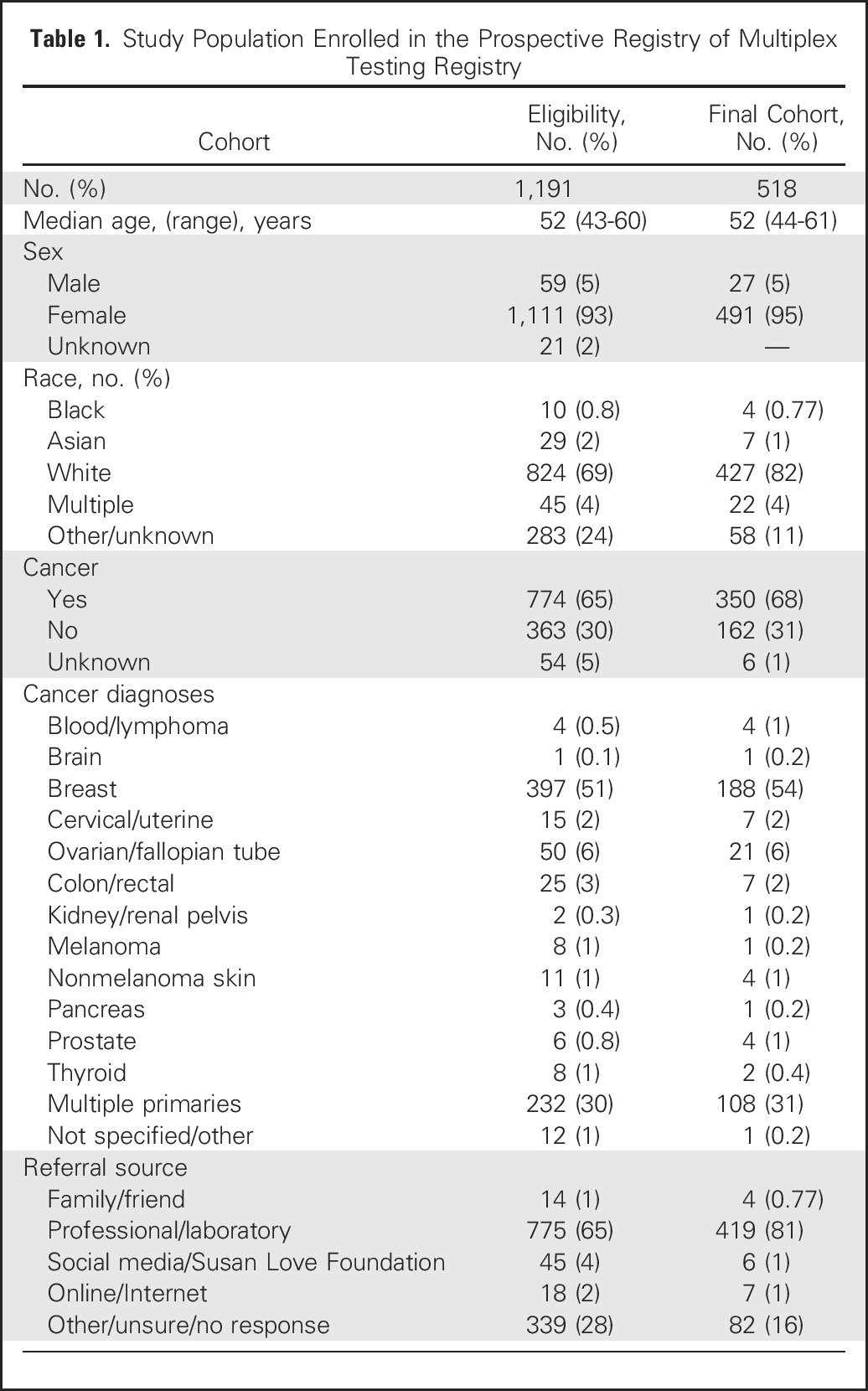
Study Population Enrolled in the Prospective Registry of Multiplex Testing Registry

**Fig 2.**
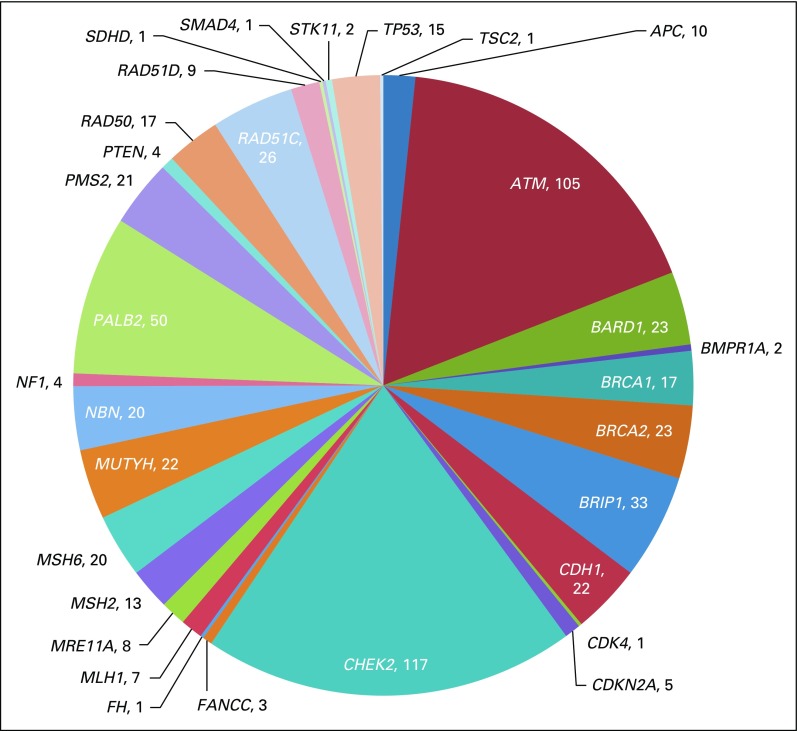
Distribution of genetic variants with multiple submissions in ClinVar by gene (N = 603).

These 518 participants reported 603 genetic variants with multiple interpretations by several commercial laboratories and/or submissions to ClinVar. Of the 518 participants included in this analysis, 165 provided information from testing done in a laboratory that does not submit to ClinVar. The most frequent gene with sequence alterations reported through PROMPT was *CHEK2* (n = 117), followed by *ATM* (n = 105) (Fig 2).

**Fig 3.**
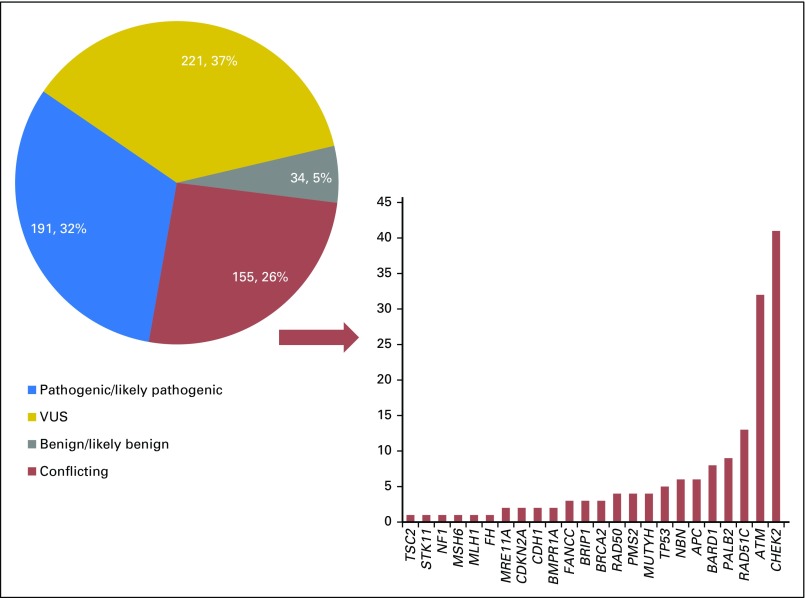
Distribution of genetic variants according to ClinVar interpretation (N = 603), and the absolute number of variants with conflicting interpretation by gene (n = 155).

Regarding the type of results according to their clinical interpretation in ClinVar, 220 (36%) were consistently classified as VUS, 191 (32%) as pathogenic/likely pathogenic, and 34 (6%) as benign/likely benign, while 155 (26%) were classified as conflicting interpretation. Among these 155, 26% of them were in *CHEK2*; 20% in *ATM*; 8% in *RAD51C*; 7% in *PALB2*; 5% in *BARD1*; 4% in *NBN* and *APC;* 3% in *RAD50, PMS2*, *TP53*, and *MUTYH*; 2% in *BRIP1* and *FANCC;* and the remainder were distributed among the other genes at approximately 1% each (Fig 3). Of the 155 discordant findings, 56 (36%) were reported as pathogenic/likely pathogenic by at least one laboratory but not by all laboratories (ie, clinically significant; Table 2).

**Table 2.**
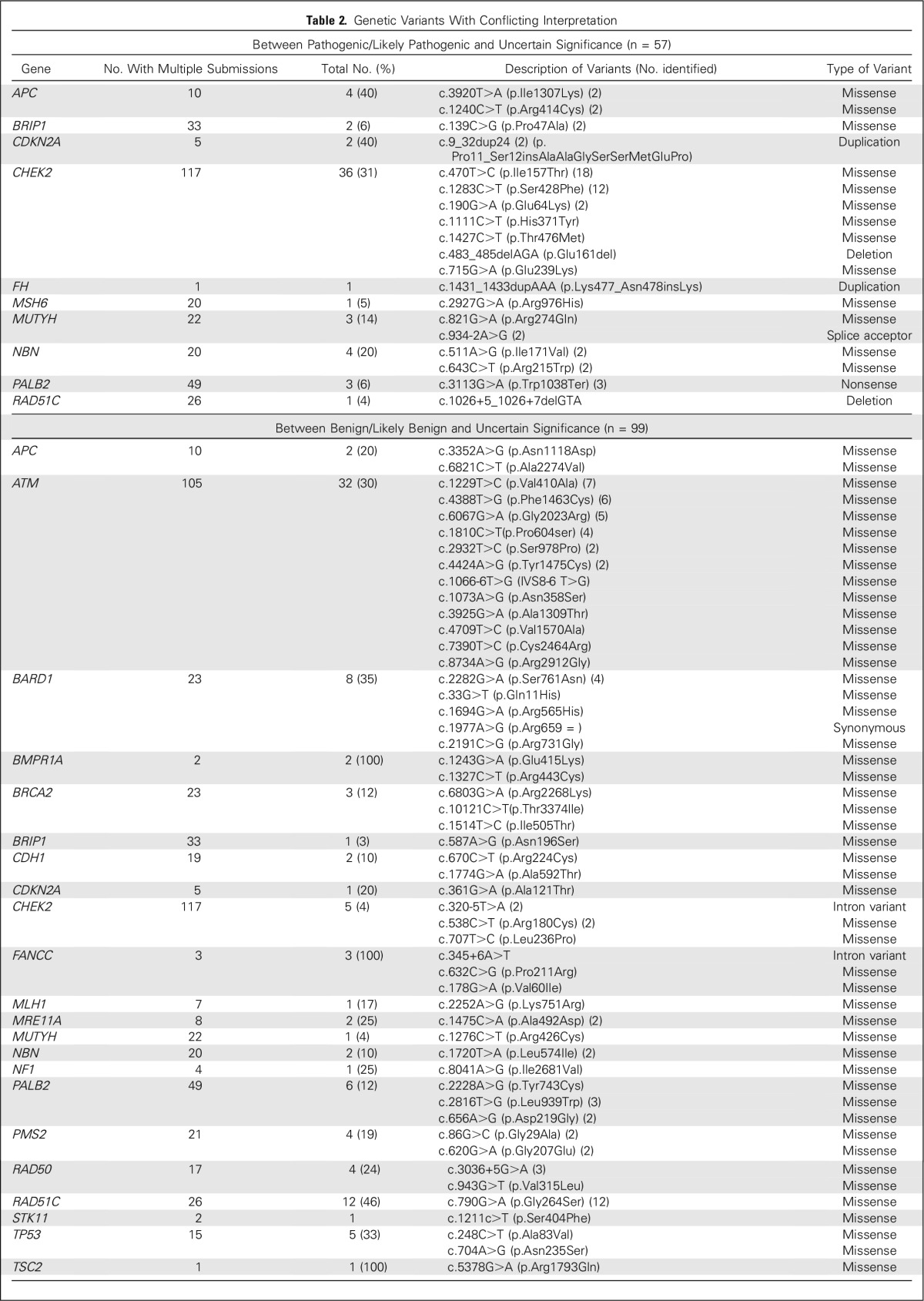
Genetic Variants With Conflicting Interpretation

**Fig A1.**
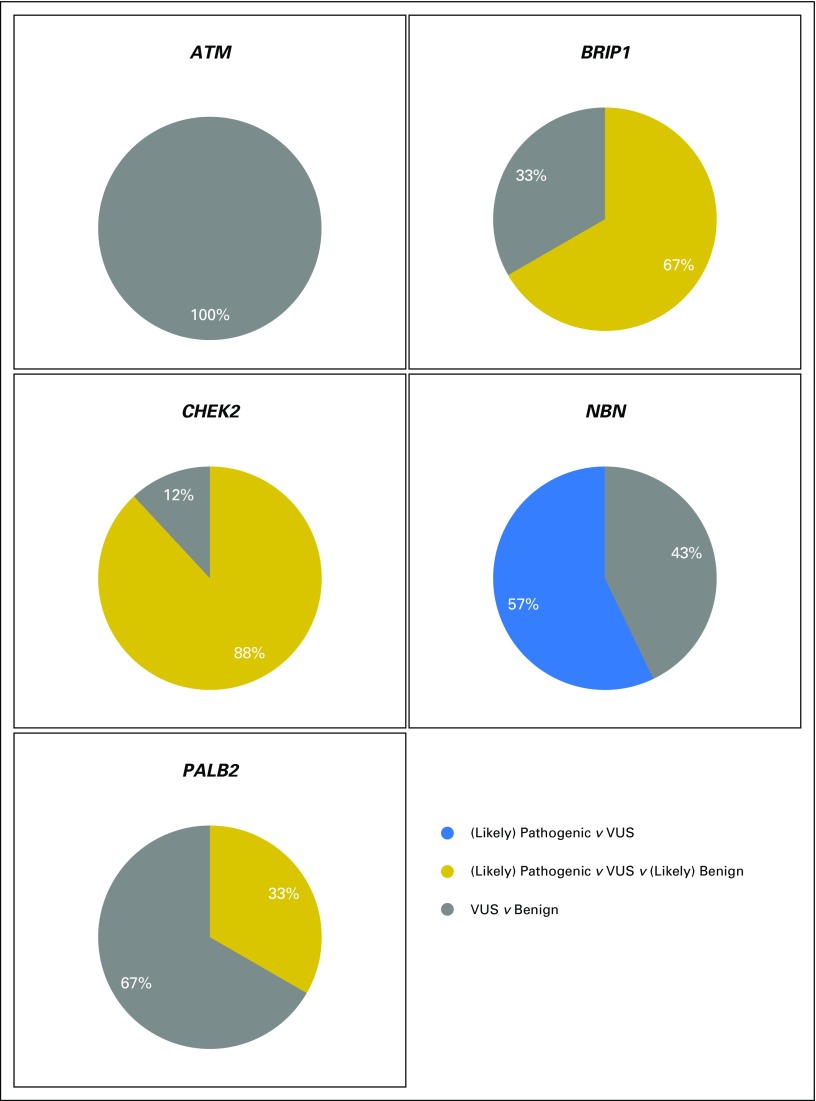
Type of conflicting interpretation by genes.

Of 117 findings with multiple interpretations for *CHEK2*, 41 (35%) were conflicting, and the majority (n = 36; 88%) would be characterized as clinically significant. Eighteen were c.470T>C (p.Ile157T) variant and 12 were c.1283C>T (p.Ser428Phe), both classified as either pathogenic/likely pathogenic or as VUS (Table 3). Other variants in this gene with a conflicting interpretation between pathogenic/likely pathogenic or VUS are described in Table 2. In addition, 32 of 105 variants (30%) in the *ATM* gene with multiple submissions were classified as conflicting. However, all of these *ATM* variants ranged from benign/likely benign or VUS (Appendix Fig A1, online only; Table 2) and, therefore, should not alter medical management. In *PALB2*, nine of 49 findings (18%) reported with multiple submissions were conflicting; one, c.3113G>A (p.Trp1038Ter), which was reported three times, was classified as either pathogenic or VUS, and the remainder were interpreted as either benign or VUS. In *BRIP1*, three of 33 findings (9%) were discordant; one of them was reported twice [c.139C>G (p.Pro47Ala)] and was classified as either likely pathogenic or VUS. In *RAD51C*, 13 of 26 (50%) findings (50%) were conflicting. Twelve of these findings corresponded to the variant c.790G>A (p.Gly264Ser), which was submitted as either benign or VUS, and one, the c.1026+5_1026+7delGTA variant, was interpreted as both likely pathogenic and VUS. In the *NBN*, six of 20 findings (30%) reported were conflicting. Two of them, c.511A>G (p.Ile171Val) and c.643C>T (p.Arg215Trp), were reported twice each, and had a two-step difference in conflicting interpretation in ClinVar (pathogenic, VUS, benign/likely benign). Other genes with conflicting interpretation are listed in Table 2.

**Table 3.**
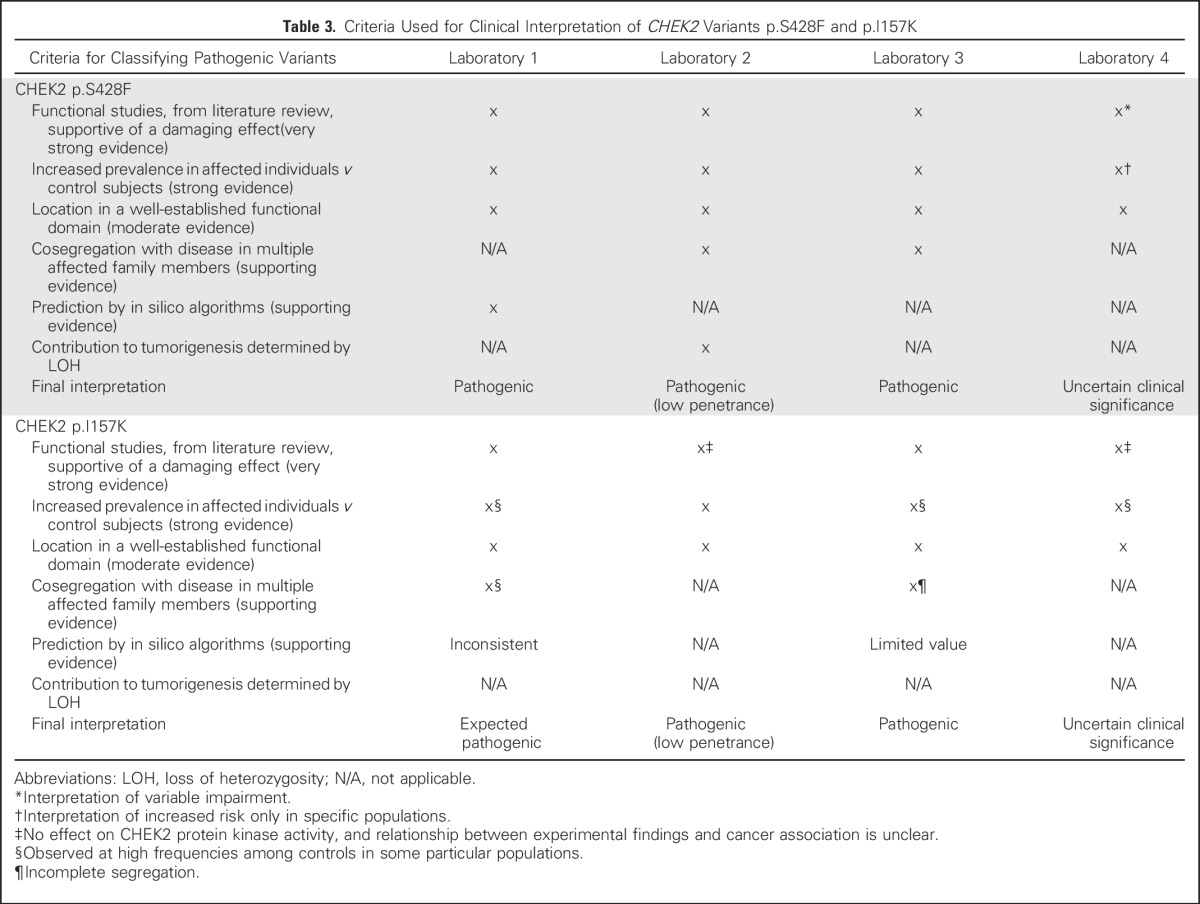
Criteria Used for Clinical Interpretation of *CHEK2* Variants p.S428F and p.I157K

## DISCUSSION

One quarter of the clinical genetic results from commercially available multiplex cancer panels and reported at the PROMPT registry had conflicting interpretations within ClinVar. Most of the variants with conflicting interpretations were in *CHEK2,* followed by *ATM*, *RAD51C*, and *PALB2*. Many conflicting interpretations are of low clinical significance because the discrepancy ranged between an interpretation of benign/likely benign or VUS; therefore, medical management should default to personal and family history. However, the identification of a VUS can cause a great deal of uncertainty for patients and providers alike and increase the risk for inappropriate medical management.^5^ For example, it is inappropriate to recommend oophorectomy based on a VUS finding alone. Of greater concern, 36% of conflicting results appeared to be clinically relevant, because they were either reported as pathogenic/likely pathogenic or as a VUS by different clinical laboratories. In this regard, *CHEK2*, *PALB2*, and *BRIP1* were most frequently identified as having discordant interpretation between these two levels of pathogenicity. As these genes are being incorporated into clinical practice as part of cancer risk assessment, tailored screening and cancer prevention recommendations, or within tumor panel sequencing for potential targeted therapy, it will be critical to standardize their curation and clinical classification of variants to provide appropriate management for mutation carriers and their families.

A strength of this study is the inclusion of individuals who underwent testing at laboratories that do not submit data to ClinVar. Our data highlight several specific variants of interest related to differential reporting. Of 117 of the *CHEK*2 findings reported in PROMPT to date, 41 (35%) were conflicting. The c.470T>C (p.Ile157Thr, I157K) and the c.1283C>T (p.Ser428Phe, S428F) variants were the most common with conflicting interpretation between pathogenic/likely pathogenic and VUS. The putative pathogenicity of I157T has long been studied. Several reports analyze its association with breast cancer risk or other tumors.^9-13^ Functional analyses of this variant have also been published^14-16^ and in silico predictions are available. The frequency of this variant in the population is 0.4% in the National Heart, Lung, and Blood Institute Exome Variant Server and approximately 5% are found in some Northern European populations.^9^ Despite these data, there was discordant interpretation of this evidence by the different clinical genetic laboratories. For instance, two laboratories found the functional analysis data compelling enough to suggest a damaging effect on protein function and to influence variant interpretation, whereas two other laboratories felt that this variant had no effect on CHEK2 protein kinase activity and the relationship between functional studies and cancer association is unclear. Only one laboratory supported the association with cancer risk as being significant, whereas the other three laboratories reported an increased prevalence in affected individuals, but also documented high frequencies observed among controls in diverse populations. All agreed that the *CHEK2* variant is located in a well-established functional domain, but only one laboratory used the supporting limited evidence provided from predictions of in silico algorithms of this variant's effect. Overall, these discrepancies in interpretation of the evidence lead to a range of clinical interpretations from VUS to pathogenic (low penetrance), likely pathogenic, and pathogenic (Table 3).

Differences in interpretation of the evidence for the pathogenicity of *CHEK2* S428F are less pronounced but still lead to conflicting reports of pathogenic, pathogenic/low penetrance, and VUS. All laboratories agree that the S428F variant is located in a well-established functional domain^16^; and all but one agree that there is evidence of an increased prevalence in affected individuals *v* controls.^9^ However, three laboratories report that functional studies are supportive of the damaging effect of *CHEK2* S428F, and one laboratory concludes that the impairment is variable. Finally, two laboratories report the literature evidence from cosegregation of the *CHEK2* variant with the disease; only one laboratory reports the prediction of pathogenicity by in silico algorithms, and another laboratory emphasizes the contribution of this variant to tumorigenesis by loss of heterozygosity (Table 3). The examples of the discrepancies involving *CHEK2* I157T and S428F also may reflect the challenges of describing a so-called low-penetrance susceptibility allele (RR < 2) in a format designed for high-penetrance alleles.^17^

Another interesting variant with conflicting interpretation between likely pathogenic and VUS is the c.139C>G substitution (p.Pro47Ala) in the *BRIP1* gene, which was observed in two unrelated participants from PROMPT. This variant was first described in 2001,^18^ when the BRIP1 protein was found to interact with BRCA1 and contribute to its DNA repair function. This variant was initially identified in an individual with early-onset breast cancer and a family history of breast and ovarian cancer; segregation analysis was not available and loss of heterozygosity in the tumor was not demonstrated. Since then, it has been reported in the literature several times as a breast or ovarian cancer susceptibility gene in affected individuals, but has also been observed in control subjects.^19,20^ Two clinical laboratories providing a summary of their clinical interpretation in ClinVar differ in their assessment of the meaning of loss of function observed in the experimental studies in regard to cancer susceptibility; one laboratory highlights that it is present in population databases, albeit as a very rare allele (0.04%); and only one endorses the supporting evidence provided by the prediction of in silico algorithms. In phase 2 of the PROMPT project, enrollment of family members will be encouraged and cosegregation analysis of this *BRIP1* variant may be undertaken, which may help provide one more piece of supporting evidence to clarify its pathogenicity.

Findings of discordant interpretation of results in genetic testing are not limited to oncologic settings nor ClinVar. Similar discrepancies also have been observed in hereditary connective-tissue disorders and are felt to be due to lack of submission of data to public databases, limited use of allele frequency data, and varying consideration of protein structure and function.^3^ In the current study, the discrepancies between commercial laboratories in interpretation of variants in cancer-related genes seem to be mostly based on differences in the interpretation of evidence. Efforts have already been initiated among laboratories to resolve these differences.^21^ The lack of a gold standard test for pathogenicity, or a noncontroversial interpretation of functional analyses, suggests that discrepant interpretation of challenging variants, particularly missense and splice-site variants, will persist for some time.

Multigene testing for cancer susceptibility is a complex endeavor characterized by challenges in curation and reporting of variants; unfortunately, this study demonstrates that conflicting interpretation of those variants may be relatively frequent. The rates of discrepant interpretation reported herein support the need for initiatives focused on harmonizing variant interpretation in the context of shared data. We encourage clinical laboratories to submit their findings to ClinVar and other public databases with relevant clinical interpretations of findings, as well as the evidence on which interpretations are based. To better identify the underlying reasons for their discrepant interpretation, laboratories could explicitly describe their weighting (from very strong to moderate or supporting) for each pathogenic criterion used for classification.^6^ This transparency in data sharing and collaboration between academic research consortia and commercial laboratories may promote strategies to standardize clinical variant curation algorithms. Ongoing consortia such as Evidence-Based Network for the Interpretation of Germline Mutant Alleles (ENIGMA), and International Society for Gastrointestinal Hereditary Tumors (InSIGHT) have demonstrated the utility of multidisciplinary collaboration to curate and reclassify submitted VUS.^22,23^ Platforms such as the Leiden Open Source Variation Database (LOVD)^24^, which allows for collection, curation, and display of phenotypes and DNA sequence variants, and the BRCA Challenge (http://brcaexchange.org/; an international effort to review and provide vetted data on *BRCA1* and *BRCA2* gene variants) are additional relevant resources. PROMPT uses both crowd-sourcing and direct participant enrollment; therefore, it also can provide a platform for cohort formation and prospective follow-up of individuals and their family members harboring genetic variants. However, at this time, health-care providers and patients need to be aware that there could be conflicting interpretations of variants and those variants may be reclassified.

Our analysis has limitations. Because PROMPT is an elective, patient-oriented registry, there may be significant ascertainment bias. For example, individuals with VUS or those who were self-aware of a variant with conflicting interpretation may have been more likely to report their variant to PROMPT, leading to an overestimation of discrepant findings. On the other hand, a significant number of test results reported by participants were not submitted to ClinVar by any laboratory and, therefore, were excluded from the primary analysis, potentially leading to a misestimate of conflicting variant interpretations. Finally, because some patients did not upload their clinical test report, it was not possible to carry out more in-depth analysis in many cases. Efforts to obtain reports from all participants are ongoing.

In conclusion, clinical interpretation of genetic testing for increased cancer susceptibility as assessed by multiplex panels hinges on accurate curation and interpretation of variants. Discrepant interpretation of some genetic variants appears to be common. Internet-based registries provide a powerful tool to collect data to inform efforts to standardize classification of genetic variants, and can play an important role in efforts to minimize potential medical harms due to false alarm or false reassurance following cancer genetic testing.
